# Scientific discovery as a combinatorial optimisation problem: How best to navigate the landscape of possible experiments?

**DOI:** 10.1002/bies.201100144

**Published:** 2012-03

**Authors:** Douglas B Kell

**Affiliations:** 1 School of Chemistry and Manchester Interdisciplinary Biocentre, The University of ManchesterManchester, Lancs, UK; 2 Biotechnology and Biological Sciences Research Council, Polaris HouseSwindon, Wilts, UK

**Keywords:** automation, epistemology, evolutionary computing, heuristics, scientific discovery

## Abstract

A considerable number of areas of bioscience, including gene and drug discovery, metabolic engineering for the biotechnological improvement of organisms, and the processes of natural and directed evolution, are best viewed in terms of a ‘landscape’ representing a large search space of *possible* solutions or experiments populated by a considerably smaller number of *actual* solutions that then emerge. This is what makes these problems ‘hard’, but as such these are to be seen as combinatorial optimisation problems that are best attacked by heuristic methods known from that field. Such landscapes, which may also represent or include multiple objectives, are effectively modelled in silico, with modern active learning algorithms such as those based on Darwinian evolution providing guidance, using existing knowledge, as to what is the ‘best’ experiment to do next. An awareness, and the application, of these methods can thereby enhance the scientific discovery process considerably. This analysis fits comfortably with an emerging epistemology that sees scientific reasoning, the search for solutions, and scientific discovery as Bayesian processes.

## Introduction

It can be of considerable value to know what makes a scientific problem ‘hard’ and why it is so, since such knowledge can, of itself, point to the best ways of attacking it. Indeed hardness and feasibility arguably represent the two chief attributes underpinning a sensible choice of a scientific problem to take on 1. Many scientific problems can be set down in a way that makes them ‘bounded’, in that there are a discrete (if large) number of possible solutions, and where the quality of the ‘objective function’ (the solution) is known or at least recognisable. Examples of such problems might be ‘find me a gene that significantly affects process X (e.g. the flowering time 2 or root length 3 in a plant)’, ‘find me a small molecule drug that at 1 µM inhibits the activity of enzyme Y by at least 50%’ or ‘find me a set of three enzymes, the removal (or modification) of each of which would lead to the maximum increase in the biotechnological production of molecule Z’.

Such problems are in fact surprisingly common in biology, even if it is possibly uncommon to set them out in this way, and the purpose of this essay is to point out that there are methods for attacking this general class of problem that are extremely effective and whose more widespread deployment would thus be of considerable scientific (and biotechnological) benefit. The general class of problem is known as a *combinatorial optimisation problem*, and is widely visualised in terms of a ‘landscape’, in which the ‘position’ in the landscape represents a candidate solution while the height represents a measure of the quality of the candidate solution at that point in the ‘search space’ or landscape of possible solutions. Finding the ‘best’ solution thus involves moving around the landscape according to an algorithm of some kind. The basic problems are (i) that the difficulty of finding ‘the best’ or ‘a good’ solution scales exponentially with the number of variables in the system, and (ii) that a genuine certainty that one has found the best *possible* solution means trying each of them (which is usually unfeasible).

Problems of this type are known as non-polynomial (NP)-hard problems (e.g. 4, 5), the number of possible solutions is typically astronomical, and thus most strategies (known as *heuristic methods* 6) simply seek a ‘good’ but not provably optimal solution.

The question of how best to move around this kind of landscape is thus equivalent in scientific discovery to determining in a principled or formal manner what is the ‘best’ experiment to do next. This is clearly a very general question, as the number of *possible* experiments is unfeasibly large; the job of the scientist is thus to choose from them effectively.

**Figure 1:**
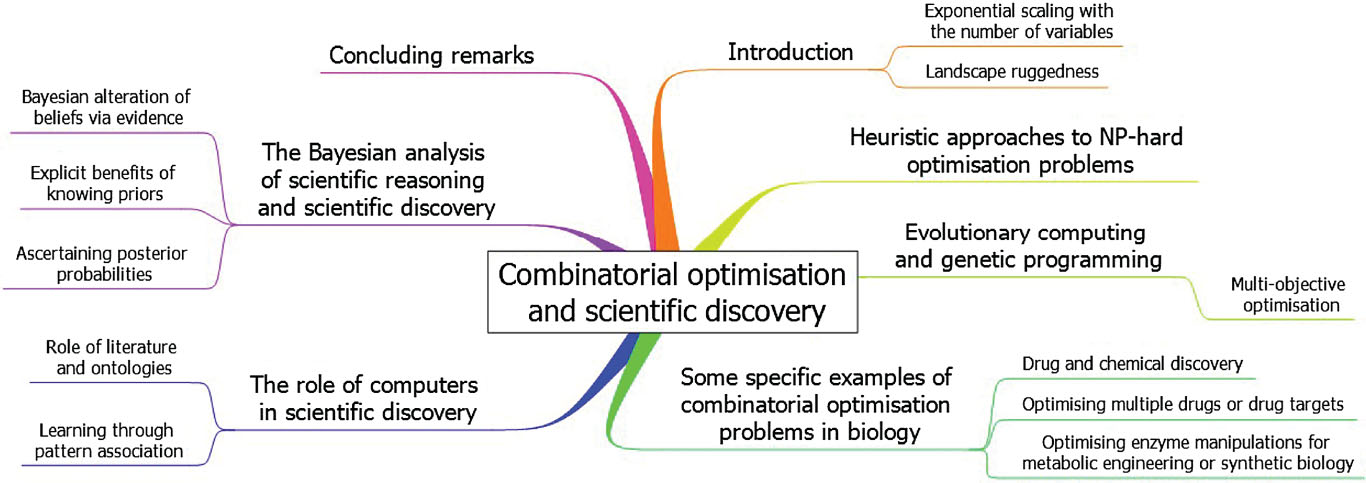
A ‘mind map’ 7 setting out the main contents of this paper. To read it start at ‘12 o'clock’ and read clockwise.

A ‘mind map’ 7 setting out the main contents of this essay is given in Fig. 1.

### Scientific problems scale exponentially with the number of variables – an example using macromolecular sequences

It is worth exploring a little this question of exponential scaling with the number of variables. To do so I choose a biological example based on aptamers. Aptamers are sequences of nucleic acids that can bind a target ligand (e.g. 8). Take the case where one seeks a DNA aptamer with the tightest binding coefficient for such a target ligand 9–11. If we consider 30mers, in which each position can be A, T, G or C, the number of possible 30mers is 4^30^, which is ∼10^18^, and even if arrayed as 5 µm spots the array would occupy 29 km^2^ 9! Clearly the number of possibilities scales exponentially with the number of bases in the nucleotide string (i.e. the variables). The lifetime of the known Universe in seconds is ∼10^17^ 12, so it is obvious that we cannot try them all.

For proteins undergoing natural or directed evolution, and using only the 20 ‘common’ amino acids, the number of sequence variants for *M* substitutions in a given protein of *N* amino acids is 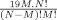 13. For a protein of 300 amino acids with changes in just 1, 2 and 3 amino acids this is 5,700, ca. 16 million and ca. 30 billion. Even for a very small protein of *N* = 50 amino acids, the number of variants exceeds 10^12^ when *M* = 10. The same combinatorial formula applies to finding the subset of k enzymes out of n that one might wish to change for some benefit; if n is 1,000 (a reasonable number for metabolism 14, 15), for *k* = 1, 2, 3, 4, 5 and 6, these numbers are 1,000, 499,500, 166,167,000, 41,417,124,750, 8.25 × 10^12^ and 1.37 × 10^15^. These numbers are already *experimentally* intractable for *k* = 3, which leads to a number of important conclusions. First, if (as is the case) most biological processes are controlled by multiple gene products, looking ‘under the lamppost’ at any number of *individual* gene products will be much less successful than seeking solutions among the much larger number of *combinations* of gene products 16. Secondly, this alone in part explains the enormous historical difficulty in developing strains by random mutation and selection for the improvement of fermentation processes. It also indicates the utility of first having a computer model of the system with which one can explore, far more effectively, the landscape of possibilities. Knowledge of where one is in the search space – of in this case sequences (strings) – can definitely help in its search (e.g. 9, 10).

### ‘Ruggedness’ reflects the nature of landscapes and the ease with which they may be searched effectively

Another issue that contributes to the difficulty of navigating these landscapes – and one can indeed make a mental picture of them as being like natural landscapes – is that they are rugged, in the sense that to access a larger peak the ‘journey’ may mean descending to a (‘fitness’) level lower than where one is presently. This concept of a fitness landscape is of course Sewall Wright's metaphor 17, and means that it is normally necessary to explore less-fit solutions en route to the discovery of a ‘better’ solution (‘reculer pour mieux sauter’ 18). In one nicely done in silico example, nearly one third of improved variants required this 19.

There are a very large number of quantitative metrics (summarised in ref. 20) for what ‘ruggedness’ means, but in general if small changes in the position in a landscape correspond to small changes in fitness while large changes in landscape position correspond to large fitness changes the landscape may be regarded as smooth. On the other hand if the two quantities (fitness and distance) are essentially uncorrelated the landscape is rugged. The basic problem is that we typically know only a tiny fraction of the landscape (and the effective structure of the landscape does depend on what kinds of moves are possible). From what we know, e.g. from the existence of divergent evolution, most landscapes are comparatively rugged, with many synergistic or epistatic interactions (i.e. the value of one variable can influence strongly the optimum value of another variable). In one example of our own, looking at the effect of changing parameters in a simple model 21, 22 of oscillations in the NF-κB signalling pathway, the effect of one parameter could be *qualitatively* different (causing oscillation frequency to go up or down) depending on the value of a second parameter 23. This is straightforwardly a consequence of the nonlinearity of most biochemical kinetic rate equations 24, together with the existence of feedback loops.

In general, cases where the effect of one variable on the behaviour of a system also depends on the value of another variable are referred to as epistatic. Such epistasis is readily observed via the co-evolution of protein residues 25 or as ‘classical’ epistasis in genetic analysis (see e.g. 16). Note too that individual residues can evolve at substantially different rates (heterotachy). Overall, the ruggedness of practical landscapes (we do not consider pathological ones such as a ‘needle in a haystack’) makes it much harder to search them effectively than if they were smooth, so many more experiments may be necessary without a good heuristic.

## Heuristic approaches to NP-hard optimisation problems

The flood of scientific data is increasing relentlessly, and this offers many novel opportunities. However, because of the effective impossibility of exploring entire search spaces experimentally for all but comparatively small problems (albeit high-throughput methods are opening up many more possibilities than were previously thought reasonable – e.g. 10), we seek good but not provably optimal solutions. As mentioned above, these are typically referred to as heuristic methods. Many effective strategies have been realised for pursuing this kind of search, which in many ways amounts to understanding and modelling the landscape itself, often in a manner that allows one to improve the *selection* of which sample to test (i.e. experiment to do) at each iteration 26, a method generally known as Active Learning (e.g. 27).

Many algorithms have been applied to these kinds of problem, and because they are essentially iterative in nature, they might be considered to be *evolutionary* in character, and indeed a major clade of optimisation strategies are known by terms such as evolutionary algorithms (EAs), evolutionary computing, evolutionary search or genetic search.

## Evolutionary computation and genetic programming

A field that has been rather explicit in its view that the solution of many scientific and technological problems is to be seen as a combinatorial optimisation problem is that of evolutionary computing (see e.g. 6, 28–31).

In evolutionary computing, as in ‘real’ (biological) evolution, there is a population of candidate solutions to a problem, each of which exhibits a level of ‘fitness’ (or more than one if the problem is multi-objective; see below). What constitutes this fitness (in terms of an objective function) is determined by the experimenter, but it is likely to include the quality of the solution and possibly also its parsimony (preference for the simplest model that is still capable of explaining all features of the system). When the fitnesses of the members of the population are evaluated there is then a selection step in which a subset of members are retained in the population and then modified, by processes akin to mutation and recombination, to produce a subsequent generation whose fitnesses are then evaluated, and so on. When the objective functions are adequately satisfied, which may mean when there is no further resource to explore the problem, the system stops and returns its optimal solution(s).

Many specific types of EA exist. One reason for this is that it can be proven (the so-called ‘no free lunch’ theorem) that which is ‘best’ depends entirely on the structure of the dataset under consideration 32, 33, with none being better than any others, including random search, when integrated over all possible datasets. However, we regard EAs as a superset of the essential kinds of strategy that can be adopted for navigating these very large search spaces of potential answers in the hope of finding ones that work adequately. Often it is not known a priori which algorithm may be best for which dataset. Trying several may be of value. Combining even ‘weak’ algorithms is known to be much more effective than choosing just a single ‘strong’ one 34.

### Multiobjective optimisation

Thus far it has been implicit that the optimisation of just a single output (e.g. an enzyme activity or the productivity of a fermentation process) was being sought. In practice, most problems are characterised by the fact that there are multiple things that one might wish to optimise. There are therefore trade-offs in that a solution optimal for one objective may be sub-optimal for another. These are known as multi-objective optimisation problems, and some are summarised in refs. 35, 36, while some of the algorithms that have been used for attacking them can be found in relevant surveys (e.g. 37).

**Figure 2:**
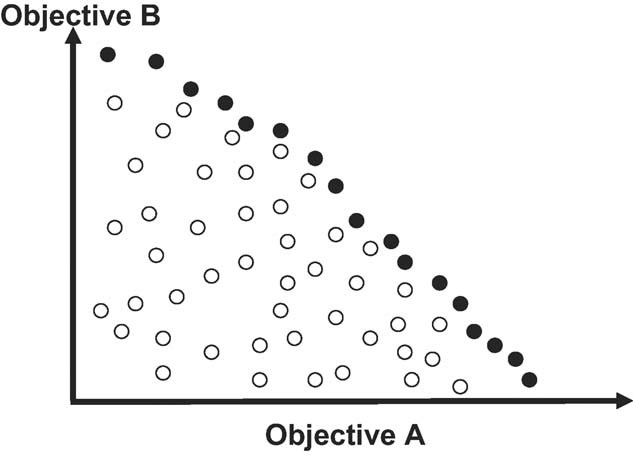
A two-objective optimisation problem, illustrating the non-dominated or Pareto front. In this case we wish to maximise both objectives. Each individual blob is a candidate solution, with the filled ones representing the approximation (based on the examples tested) to the Pareto front.

These trade-offs are usually expressed in terms of the so-called Pareto or ‘non-dominated’ front, represented by solutions that are best in terms of at least one objective and not worse in terms of any other. The Pareto optimal set of solutions (individuals) consists of all those that it is impossible to improve in any objective without a simultaneous worsening in some other objective, and is illustrated diagrammatically in Fig. 2.

Most of the examples we are looking at here are also implicitly multi-objective in nature, e.g. in terms of optimising a protein we might wish it to have a very high *k*_cat_ but also to survive at elevated temperatures or in high concentrations of solvent (the choosing of which is itself a combinatorial and multi-objective problem 38), which might themselves cause *k*_cat_ to vary slowly over time. A very common set of problems is represented by those for which a ‘better’ solution is also a more expensive one, and thus cost is typically one criterion of the (multi-)objective function. Typically the choice of solutions from the Pareto front is at the behest of the experimenter, and for this reason we shall largely ignore multi-objectivism since our focus is on the combinatorial issue. One point worth making, however, is that the more objectives one includes the more nearly does the search approach a random search.

## Some specific examples of combinatorial optimisation problems in biology

The aptamer example given above is formally equivalent to any problem of ‘directed protein evolution’, protein structure prediction or folding. In addition, it is worth highlighting the following problems as best approached via combinatorial optimisation: drug discovery; optimising cocktails of known drugs; identifying targets for metabolic engineering. I ignore other quite general NP-hard problems such as ‘clustering’ where there can be many objects and variables (e.g. 39–41).

### Drug and chemical discovery

Drug discovery is a tricky and costly process 42, and nowadays usually involves the search for a molecule that can bind tightly to (and inhibit) a chosen molecular target.

However, because of the multiple valencies of carbon, and its ability to bind with many other multivalent atoms such as N and O and the monovalent H, Cl, Br and F, the number of possible molecules with a given number of such atoms is enormous – tens of millions even for molecules with molecular masses below 160 Da and atom numbers of C, N, O and F up to 11 43 and ignoring stereoisomers. Reymond and colleagues have recently extended the analysis to the ca. 977 million compounds with 13 atoms of C, H, N, O, S and Cl 44 (and see http://www.dcb-server.unibe.ch/groups/reymond/). Few of these compounds have been made, and with a realistic drug discovery space of maybe 10^60^ compounds 45 most will not. Indeed even most simple heterocycles have not been explored at all.

An emerging solution to this is to ‘evolve’ molecules with desirable properties by bringing together fragments that themselves are not optimal – so-called fragment-based drug (or lead) discovery (e.g. 46). In this case, discovery proceeds in a manner analogous to that of the evolutionary search described above, where each population member is a molecule representing a candidate solution. The fitness (e.g. binding strength) of the various solutions is evaluated and then solutions mutated and/or recombined to make different and often larger molecules (since these will tend to have more atoms that can bind to the target). In each generation only a few hundred molecules are typically used, rather than the tens of thousands or even millions available in pharmaceutical drug libraries. Candidate solutions can be screened *virtually* by performing a quantitative structure-activity analysis at each step, i.e. providing a computer model that effects a mapping between known structures and their fitnesses, then assessing the quality of potential leads in silico. This is made considerably easier by the online listings of huge numbers of commercially available molecules, e.g. in the ZINC database http://zinc.docking.org/, chosen subsets of which from the virtual screening can then be tested experimentally. (A similar approach using virtual screening with aptamers was extremely successful 9.) Note too that other aspects may need to be optimised, e.g. the likelihood that such molecules will be substrates for cellular drug transporters (e.g. 47–49).

The discovery of an individual substance – here a pharmaceutical drug – from a potentially gigantic catalogue of possible substances clearly requires effective means of searching for it. A related problem is finding appropriate mixtures or cocktails from a potentially large set of combinations of known substances.

### Optimising multiple drugs or drug targets

There is increasing recognition that to be effective (whether singly or in combination), pharmaceutical drugs must affect multiple steps simultaneously 50–52. This follows in part from the facts that (i) the flux through networks is very rarely controlled by a single step as this is a systems property 53, and (ii) biological systems have tended to evolve towards robustness (if modifying just one parameter causes death then evolution soon selects against such a cell or organism). However, by and large we still lack good biochemical network models 54, 55 over which to reason.

Clearly the combination formula given above shows that the number of combinations scales exponentially with the number of real and possible choices one can make, and if there are n separate candidate drugs the possible number if all may be used is 2^*n*^ (each is either used or not used).

Again, a number of recent examples (e.g. 52, 56) show how a heuristic search of combinations of drugs with ‘known’ individual targets can swiftly lead to effective solutions, often involving synergy such that lower concentrations of potentially toxic individual components can be used.

Optimising such mixtures is effectively the same as optimising the components of a medium for improving the productivity of a fermentation for biotechnology, and Weuster–Botz and colleagues have developed such a strategy to great effect (e.g. 57). The same applies to the optimisation of any ‘recipe’ or process that has a number of possible components and steps whose nature and/or properties may be varied.

### Optimising enzyme manipulations for metabolic engineering or synthetic biology

Optimising biotechnological processes in the modern era is probably best seen to involve choosing the enzymes that most need modification and then optimising them individually by directed evolution 58. Finding a simple combination of enzymes to manipulate for improving a desirable trait is formally equivalent to finding a (small) set of drug targets, and is certainly a combinatorial optimisation problem, and it does seem to be the case that a small number of carefully chosen targets can often have large effects (e.g. 14, 59–61). Historically we lacked both the necessary models 53 and the molecular biology techniques, and progress was both slow and empirical 62. In particular, if we need to manipulate just four enzymes out of say 1,000 (a typical number for microbial metabolic networks 15, 63, 64), the number of combinations is about 41 billion, somewhat beyond the typical abilities of a wet lab. However, such a number can be tested in silico in a comparatively short time (and, like most such analyses 20, the test can be parallelised perfectly). This explains the need for having a half-decent in silico model with which to work and make predictions.

In a particularly nice example from ‘white’ or industrial biotechnology, this is exactly what Sang Yup Lee and coworkers did 14 for improving (considerably) the production of valine in *Escherichia coli*, first exploring in silico the ∼10^8^ search space to find three enzymes from ca. 1,000 to manipulate, and then doing so experimentally. Broadly similar strategies have proved efficacious for a variety of other products 58.

## The role of computers in scientific discovery

The treatment of many or most scientific problems as combinatorial problems is to be seen as a subset of a broader field that seeks to formalise the use of computers or ‘artificial intelligence’ in scientific discovery (e.g. 26, 65, 66), with the metric of whether such results are ‘human-competitive’ 67 being at least one measure of success. Indeed, every experiment consists of various steps with different properties that can be varied independently and this is why designing an experiment is a combinatorial problem.

### Learning can be effected through association of patterns

The present kind of principled reasoning approach usually involves some kind of association or pattern matching analysis based on data mining techniques, and is to be seen as a kind of inductive reasoning 68 in which paired data are used as inputs to a learning system from which more general rules are expected to emerge 69. Starting with the DENDRAL system 28–31 that sought implicitly to learn rules for molecular decomposition in mass spectrometers, and thereby the identification of molecules from their mass spectra (‘from spectrum to structure’ 74), a number of computer-based scientific discovery systems have been proposed. A couple of reviews may be cited (e.g. 65, 75), and I list some of the specific systems in Table 1. A number of these are iterative, and even closed-loop (requiring no human intervention) whereby the results of the analysis lead to the proposal and performance of the next ‘wet’ experiment in a series (active learning – see above) as the system learns to optimise what it seeks to discover.

**Table 1:** Some of the systems that have been devised for automating the process of scientific reasoning

Name	Field of endeavour	Selected references
Dendral (and meta-dendral)	Mass spectrometric identification of molecules	70, 72, 73
Bacon	Thermodynamics, heat capacity and heat flow	76
Fahrenheit	Electrochemistry	77
Not named	Quantum control of chemical reactions	78–80
The robot scientist	Yeast metabolism	27, 28–31
The robot chromatographer	Chromatographic optimisation	85, 86
Not named/Eureqa	Dynamics	87, 88
Clade	Aptamer evolution	9–11,75,89

### The role of the scientific literature and ontologies

The means of capturing, encapsulating and transmitting knowledge lies at the heart of science, and from the computational point of view the literature remains an imperfectly accessed resource 90, 91. It is not even easy to answer well the question ‘which is the best paper for me to read next?’. More importantly, it is the general concept of semantics that differentiates raw text from text with meaning (e.g. 28–31). Nowadays it is considered that the use of RDF triples for simple relationships and for more complex ones the more full-blown ontologies – of which the Gene Ontology 96 (http://www.geneontology.org/) is probably the best known to biologists – is the most effective means with which to imbue text with meaning as part of the general computational area known as text mining (e.g. 92, 97). Since much knowledge can be encoded as graphs, the Systems Biology Markup Language 98 – which is designed for describing them in a principled manner – seems a natural means of doing this 99, especially since it can reference its own ontology directly (e.g. 15, 100–102). This involves in part finding the literature that provides the *evidence* for a particular pathway; the converse problem (‘given the literature, construct the pathway’) is an important direction, but considerably harder.

One major difficulty with conventional ontologies is that they do not easily deal with (i.e. reconcile) contradictory statements; for this, and for inferencing more generally, some kind of weighting system is required.

## The Bayesian analysis of scientific reasoning and scientific discovery

A comparatively recent development (e.g. 68, 103–108) is the recognition that the application of the methods of Bayesian inference provides a straightforward and natural means of understanding the relative roles of old and newer evidence in the development of theory and belief. Certainly, one can hardly deny that science and other endeavours involve a continuing series of inferences based on incomplete data. In the classical form (e.g. 109–113), Bayes' rule (more accurately the rule of Bayes, Price and Laplace 107) simply states that a new set of observations (‘evidence’) of *B* regarding two events *A* and *B* adds to or otherwise our belief in a particular point of view of *A* according to the Bayes formula


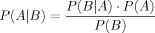


where *P*(*A*|*B*) is the ‘posterior’ or conditional probability of *A* given *B*, *P*(*B*|*A*) is the conditional probability of *A* given *B* (also known as the likelihood), *P*(*A*) is the ‘a priori’ or prior probability of *A* in absence of the extra knowledge provided by measurement of *B*, while *P*(*B*) is the prior (or marginal) probability of *B*. (In many experimental set-ups, *A* is to be seen as a ‘cause’ of the experimentally observable ‘effect’ *B*.)

### Ascertaining posterior probabilities

To see how this works, imagine members of two tribes (let us call them Hawks and Jets) who inhabit an island. There are 1.5 times as many Hawks as Jets. All Hawks wear blue tunics but for Jets 50% wear blue tunics and 50% wear brown tunics. If you meet a person wearing a blue tunic what is the probability that they are a Jet?

If *P*(*A*) is the a priori probability of being a Jet, it is 0.4. *P*(*B*), the a priori probability of wearing blue, is 0.6 + (0.5 × 0.4) = 0.8. *P*(*B*|*A*), the probability of wearing blue if you are a Jet is 0.5. Application of the Bayes formula therefore gives the requested probability *P*(*A*|*B*), the probability of being a Jet given that you are observed to be wearing blue, as 0.5 × 0.4/0.8 = 0.25. These binary outcomes can be put into a tabular form, where the fraction of ‘Blue Jets’ to ‘total Blues’ is clearly 20/80 = 0.25.

**Table:** 

	Hawks	Jets	Total
Blue	60	20	80
Brown	0	20	20
Total	60	40	100

### Explicit benefits of knowing priors

Bayesian analysis also allows one to take priors into account in a way that so-called frequentist statistics do not. In binary outcomes (true/false) in diagnostic tests e.g. for disease we can have four outcomes: true positives (TPs), false positives (FP), true negatives (TN) and false negatives (FN). The sensitivity of the test (see e.g. 114) describes its ability to spot positive results (i.e. the person tested has the disease for which the test is diagnostic):


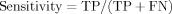


While the specificity determines the ability of the test to detect negative results (i.e. correctly to identify those who do not have the disease)


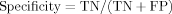


Suppose one has developed a diagnostic test for a disease that has a sensitivity of 99% and a specificity also of 99%. On most grounds this might seem an excellent test, but this ignores the priors. Imagine now a (real) population in which only 1% of the individuals in the population actually has the disease, which is not unreasonable.

If *A* is the disease, and *B* a positive result, application of the Bayes formula gives


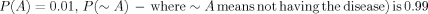



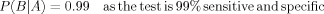


and *P*(*B*) = 0.99 × 0.01 + 0.99 × 0.01 = 0.0198, so *P*(*A*|*B*), the probability of having the disease given a positive result, is 0.99 × 0.01/0.0198, which is only 0.5. So despite the very high sensitivity and specificity of the diagnostic, the very low *prevalence* of the disease (the prior), means that actually the test (and probably any individual test…) is rather poor.

The equivalent table (rounded to integers for 1,000 tests) is as follows:

**Table:** 

	Diseased	Not diseased	Total
Disease predicted	10	10	20
Disease not predicted	0	980	98
Total	10	990	1,000

### A Bayesian view of the alteration of scientific beliefs based on new evidence

More recently, Bayesian thinking has been applied in terms of how new evidence alters our degrees of belief about something, as part of the scientific process. As recently phrased by Tenenbaum et al. 108,

“Background knowledge is encoded through a constrained space of hypotheses *H* about possible values for the latent variables, candidate world structures that could explain the observed data. Finer-grained knowledge comes in the ‘prior probability’ *P*(*h*), the learner's degree of belief in a specific hypothesis *h* prior to (or independent of) the observations. Bayes's rule updates priors to ‘posterior probabilities’ *P*(*h*|*d*) conditional on the observed data *d*:


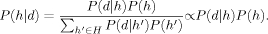


The posterior probability is proportional to the product of the prior probability and the likelihood *P*(*d*|*h*), measuring how expected the data are under hypothesis *h*, relative to all other hypotheses *h*′ in *H*.”

Thus, in a series of experiments in an experimental program, one chooses an experiment based on some background knowledge and performs the experiment. The results of the experiment add to the background knowledge for the next experiment that one chooses to do, and so on. Thus, in the Bayesian view, the priors represent the existing knowledge from previous experiments, that are clearly a function of how much of the search space has already been searched and to what effect. The posterior probabilities are updated via the new set of data, and the new ‘knowledge’ is encoded in the degree of belief.

Thus, if we take a protein directed evolution example, where one is seeking to find out which kinds of sequences (and/or structures) exhibit a high *k*_cat_ for a suitable enzyme activity, the background knowledge is represented by any known enzymes or sequences associated with an activity of interest (that may be a catalytic activity similar but not identical to that being sought). The prior probabilities are encapsulated in any known sequence-activity relationships previously existing that lead one to test some related ones in the experiment at hand. Following the new experiments (that measure pairs of sequences and activities), the posteriors, that are the priors for the next experiment, have to be readjusted since the new data modify the previous structure-activity relationship.

This seems to translate naturally into the recognition that many scientific problems are combinatorial problems with a large but effectively bounded search space and as we improve our knowledge of the search space we thereby increase our degree of belief in any more general properties of that search space (in the previous example a sequence-activity relationship, represented in ref. 9 via a so-called ‘random forest’. In another example, Bayesian methods can usefully be applied to the analysis and ranking of network or systems biology models that start with observables and seek the underlying parameters or causes (e.g. 28–31).

## Concluding remarks

Scientific discovery and reasoning can usefully be seen as an iterative cycle linking more inductive phases of hypothesis generation and more deductive activities involving the testing of the hypotheses so generated 69. However, this leaves open the question of the means for optimising the former phase. This is the subject of the present work, where I have set out the view that this is best seen as a combinatorial optimisation problem over a search space of possible experiments.

To this end, I have provided a series of examples in which scientific problem domains are easily recognisable effectively as combinatorial optimisation problems, where a very large search space admits a considerably smaller solution space of ‘adequate’ answers. If one accepts that *any* scientific problem has a number of solutions that is much smaller than the ‘possible’ numbers of experiments that might seek them, the same holds true more generally. Since searching a combinatorial landscape computationally (i.e. in silico) is considerably more rapid and efficient than is performing ‘real’ experiments at every point, it is clear that we need much more effective models of biology than we have today 54. This impels us to create and to analyse them as part of the iterative process of scientific discovery.

## Abbreviations

EA: evolutionary algorithm
FN: false negatives
NP: non-polynomial
FP: false positives
TN: true negatives
TP: true positives
